# Negative Pressure Wound Therapy Versus Conventional Dressings After Debridement for Necrotizing Fasciitis or Gas Gangrene: Impact on Hospital Stay and Wound Closure Methods

**DOI:** 10.7759/cureus.109563

**Published:** 2026-05-24

**Authors:** Juri Sugiyama, Yasuhiko Takegami, Satoshi Muto

**Affiliations:** 1 Department of Orthopaedic Surgery, Nagoya Medical Center, Nagoya, JPN; 2 Department of Orthopaedic Surgery, Nagoya University Graduate School of Medicine, Nagoya, JPN

**Keywords:** gas gangrene, length of stay, necrotizing soft tissue infections, negative pressure wound therapy, surgical debridement, wound closure techniques

## Abstract

Background: The efficacy of negative pressure wound therapy (NPWT) in managing severe soft tissue infections (SSTIs), including necrotizing fasciitis and gas gangrene, remains controversial. This study aimed to compare clinical outcomes of NPWT versus conventional dressings following surgical debridement in patients with these conditions.

Methods: We conducted a multicenter retrospective cohort study across 11 tertiary hospitals (2015-2024). We included adult patients with necrotizing fasciitis or gas gangrene of the lower extremity and trunk who underwent surgical debridement. Patients were divided into two groups based on postoperative wound management: NPWT (n = 28) and conventional dressings (n = 46). The primary outcome was total hospital length of stay. Secondary outcomes included the number of debridements, wound closure method, and functional decline at discharge.

Results: We analyzed 74 patients. Hospital stay was significantly longer in the NPWT group versus the conventional dressing group (mean = 80.36 vs. 43.17 days; mean difference = 37.19 days; p < 0.001). Patients in the NPWT group also required more debridement procedures (mean = 2.64 vs. 1.70; p = 0.008) and were significantly more likely to undergo split-thickness skin grafting (57.1% vs. 13.0%; p < 0.001), whereas primary closure was more common in the conventional group (17.9% vs. 50.0%). Ambulatory functional decline was not significantly different between the groups.

Conclusion: In the management of necrotizing fasciitis or gas gangrene, NPWT was associated with significantly longer hospital stay and more complex wound closure procedures, without clear benefits in functional recovery at discharge. These findings suggest that while NPWT may be useful for local wound management, its routine use should be approached with caution, and careful patient selection remains essential.

## Introduction

Severe soft tissue infections (SSTIs), including necrotizing fasciitis and gas gangrene, are rapidly progressive and life-threatening conditions caused by toxin-producing bacteria [1,2]. Necrotizing fasciitis is characterized by rapidly spreading infection of the superficial fascia with relative sparing of overlying skin and underlying muscle in its early stages, whereas gas gangrene predominantly involves deep muscle tissue with extensive necrosis and gas formation [3]. Both conditions are associated with severe pain, systemic toxicity, and high mortality rates that require immediate surgical intervention to obtain optimal survival outcomes [4]. As the lower extremities are the most common anatomical site for such infections, optimizing wound management strategies in these cases remains a crucial clinical challenge [5].

The primary goal in treating SSTIs is to control infection through urgent and aggressive surgical debridement combined with broad-spectrum antibiotics and intensive supportive care [6]. However, the management of extensive open wounds that remain after surgical intervention presents a major clinical challenge. While research on wound management methods, including negative pressure wound therapy (NPWT), has advanced considerably, standardized guidelines for post-debridement wound care in SSTIs remain to be established, particularly concerning the optimal use and timing of advanced therapies such as NPWT [7-9].

Traditional post-debridement wound care relies on daily dressing changes, such as wet-to-dry dressings, to maintain wound cleanliness and promote granulation tissue formation. However, these methods are often time-consuming, painful for patients, and require prolonged healing periods. In many cases, delayed wound closure becomes necessary, which involves skin grafts or flap reconstructions once infection is fully controlled [10]. The burden of frequent dressing changes and extended treatment duration has led clinicians to seek alternative wound management strategies that might accelerate healing and reduce overall treatment complexity.

NPWT has emerged as a promising alternative for managing complex wounds. NPWT applies controlled negative pressure to the wound bed through a sealed dressing system, which is believed to enhance wound healing by promoting granulation tissue formation, reducing edema, and improving local blood flow [11-13]. Several studies have reported favorable outcomes with NPWT in various wound types, including diabetic ulcers, pressure sores, and post-surgical wounds, by demonstrating reduced healing time and improved closure rates compared to conventional dressings [14,15]. Although NPWT has been increasingly adopted for complex infected wounds after debridement, evidence supporting its routine use in necrotizing soft-tissue infections remains limited and inconsistent. A large-scale randomized study for severe necrotizing fasciitis showed no significant differences in mortality and amputation rates between conventional therapy and NPWT [16], raising questions about the clinical benefits of NPWT in this specific patient population.

Given the conflicting evidence and limited data on NPWT outcomes in SSTIs, this study aimed to compare the clinical effectiveness of NPWT versus conventional wound dressings in patients with SSTIs of the lower extremity and trunk. Specifically, we sought to evaluate differences in hospital length of stay, wound closure methods, functional outcomes, and treatment-related complications. Understanding these outcomes is crucial for developing evidence-based guidelines for post-debridement wound management in this high-risk patient population.

## Materials and methods

Study design

We conducted a multicenter retrospective cohort study of patients with SSTIs of the lower extremity treated between January 2015 and December 2024 at 11 tertiary hospitals affiliated with the Trauma Research Group of our university. The SSTIs included necrotizing fasciitis and gas gangrene. Our registry prospectively captures all emergency and orthopedic admissions and serves as the primary data source. Institutional review board approval was obtained from each participating center, and the study was performed in accordance with the Declaration of Helsinki.

Patient selection

Patients aged 18 years or older with SSTIs of the lower extremity and trunk requiring surgical debridement were included in this study. SSTIs were defined as necrotizing fasciitis or gas gangrene confirmed by clinical presentation, imaging findings, and intraoperative findings. Inclusion criteria were (1) diagnosis of necrotizing fasciitis or gas gangrene, (2) surgical debridement performed within 24 hours of diagnosis, and (3) availability of complete medical records, including wound management details. Exclusion criteria included (1) patients who died within 48 hours of admission, (2) those with incomplete follow-up data, (3) patients with concurrent malignancy, and (4) those who underwent primary amputation without attempt at limb salvage.

**Figure 1:**
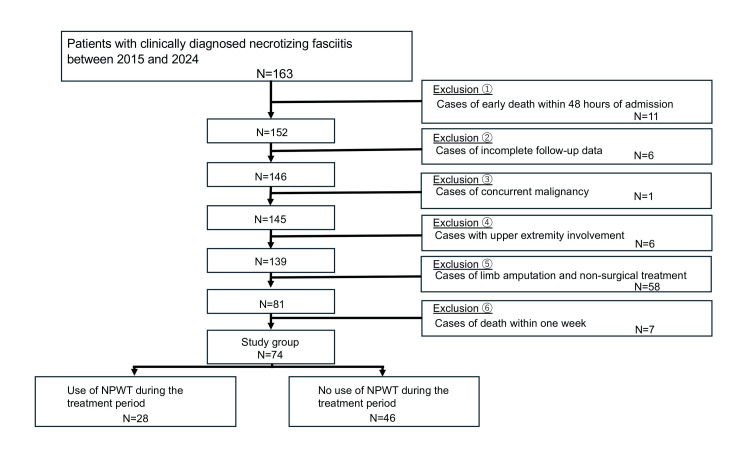
Flow diagram of patient selection and group allocation. NPWT: negative pressure wound therapy.

The patient enrollment process is illustrated in Figure 1. Between January 2015 and December 2024, 145 patients were consecutively registered with a diagnosis of necrotizing fasciitis or gas gangrene. Six patients were excluded because the infection involved the upper extremity. A further 58 patients were excluded because they either underwent primary limb amputation or received only conservative (nonsurgical) management, including those treated with palliative intent. Additionally, seven patients who died within one week of admission were excluded, as this precluded complete follow-up or assessment of the studied wound management strategies. This screening process resulted in a final study cohort of 74 patients who were divided into the NPWT group (n = 28) and the conventional dressing group (n = 46).

Data collection

A comprehensive set of variables was extracted for each patient from the registry and individual electronic medical records.

Demographics and Medical History

Patient demographics included age, sex, body mass index, smoking and alcohol history, illicit drug use, living environment (home vs. nursing facility), and social support status (e.g., public assistance). Comorbidities were systematically assessed using the Charlson Comorbidity Index (CCI) and included hypertension, myocardial infarction, congestive heart failure, peripheral arterial disease, cerebrovascular disease, dementia, chronic pulmonary disease, collagen disease, steroid or other immunosuppressive therapy, atopic dermatitis, peptic ulcer disease, liver disease, diabetes mellitus (with or without insulin), hemiplegia, renal impairment, solid tumor, leukemia, lymphoma, metastatic cancer, and HIV/AIDS [17]. Regular medications, including anticoagulants, antiplatelets, immunosuppressants, steroids, and psychotropics, were also documented.

Premorbid ambulatory ability and ambulatory function at discharge were assessed using a six-point ordinal scale defined as follows: 0 = independent ambulation; 1 = cane-assisted or supported walking; 2 = walker or rollator use; 3 = wheelchair; 4 = bedridden; and 5 = unknown. Walking ability decline was defined as an increase of at least one level (i.e., deterioration) in this score at discharge compared to the premorbid baseline status. Patients with an unknown ambulatory status (score 5) at either assessment point were excluded from analyses involving walking ability decline.

Admission Vital Signs and Laboratory Parameters

On admission, vital signs were systematically recorded, including heart rate, systolic and diastolic blood pressures, mean arterial pressure, respiratory rate, oxygen saturation, body temperature, and level of consciousness (Glasgow Coma Scale).

Laboratory parameters obtained at admission included complete blood count with differential, coagulation studies (activated partial thromboplastin time, prothrombin time, international normalized ratio, fibrin degradation products, D-dimer), serum electrolytes (sodium and potassium), inflammatory markers (C-reactive protein, procalcitonin), metabolic parameters (creatinine, albumin, blood glucose, glycosylated hemoglobin), and organ function markers (aspartate aminotransferase, creatine kinase, lactate dehydrogenase, blood urea nitrogen, serum lactate).

The Laboratory Risk Indicator for Necrotizing Fasciitis (LRINEC) score was calculated for every patient on admission [18]. With the use of the LRINEC score, patients were stratified into three risk categories: low (LRINEC score ≤5), moderate (LRINEC score = 6-7), or high (LRINEC score ≥8) risk for necrotizing soft tissue infections.

Intensive Care and Surgical Intervention Data

Data pertaining to intensive care unit (ICU) management and surgical interventions were systematically collected. ICU-related variables included duration of ICU stay, requirement for postoperative mechanical ventilation, use of vasopressors, and hemodynamic support measures. Surgical intervention details encompassed the timing of initial debridement, operative duration, estimated blood loss, extent of tissue excision, and the total number of debridement procedures required throughout the hospital course. Additional procedural data included the need for fasciotomy, amputation rates, and any surgical complications encountered during the treatment period.

Initial surgical management

All patients included in the study underwent emergency radical debridement under general anesthesia as the primary treatment intervention. During this initial procedure, all nonviable skin, subcutaneous tissue, and fascia were systematically excised until healthy, bleeding tissue margins were reached. Following complete excision, wounds were thoroughly irrigated with 0.9% saline solution. Wounds were initially left open to allow for assessment and planned repeat debridements at intervals of 24-72 hours until both clinical and microbiological control of the infection was achieved. The decision for repeat debridement was based on clinical examination findings, laboratory markers of infection, and intraoperative assessment of tissue viability. Key operative details recorded for each surgical procedure included the anatomical extent of tissue excised, operative duration, estimated blood loss, and the total number of subsequent debridement procedures required per patient.

Post-debridement wound management

Following initial and any subsequent debridements, patients were managed using one of two wound management strategies based on clinical judgment and institutional preferences. Given the retrospective study design, standardized treatment protocols were not applied, and the choice between NPWT and conventional dressings was determined by individual surgeon preference rather than predetermined criteria.

Conventional Dressing Group (Non-NPWT Group)

Patients in this group received daily dressing changes using wet-to-dry gauze or alginate dressings. Dressing selection was individualized based on wound characteristics and drainage volume.

NPWT Group

Patients in this group received NPWT using polyurethane foam dressings connected to either a RENASYS GO® (Smith & Nephew, London, UK) or V.A.C.® (Solventum, Eagan, MN) NPWT system. Continuous negative pressure of -125 mmHg was typically applied, though this was reduced to -80 mmHg for wounds considered fragile or when excessive bleeding was observed. NPWT dressings were generally changed every 48-72 hours or more frequently if clinically indicated.

For both groups, device-related complications (including bleeding, excessive pain, or retained foam in the NPWT group) and significant deviations from the prescribed dressing protocol were systematically documented. The assigned dressing type was discontinued in both groups once the wound bed showed greater than 90% healthy granulation tissue or was otherwise deemed ready for definitive closure based on clinical assessment.

Antimicrobial therapy

Empiric broad-spectrum antimicrobial therapy was initiated immediately after obtaining appropriate cultures from blood, wound tissue, and any purulent material. The standard empiric regimen consisted of either meropenem plus clindamycin or piperacillin/tazobactam to provide coverage against Gram-positive, Gram-negative, and anaerobic organisms commonly associated with necrotizing soft tissue infections. Vancomycin was added empirically if methicillin-resistant *Staphylococcus aureus* infection was suspected based on clinical risk factors or institutional epidemiology. Once culture and antimicrobial sensitivity results became available, therapy was de-escalated or modified accordingly to target the identified pathogens. Intravenous antibiotics were continued for a minimum of 14 days or until clinical improvement was shown by normalization of inflammatory markers (C-reactive protein <5 mg/L) and the patient remained afebrile for at least 48 hours. Following completion of the intravenous course, treatment was typically transitioned to oral antibiotics to achieve a total antimicrobial duration of four to six weeks, depending on clinical response and wound healing progress. All antibiotic-related adverse events, including allergic reactions, organ toxicity, and treatment discontinuations, were systematically documented throughout the treatment period.

Criteria for definitive wound closure

Definitive wound closure was considered when the following criteria were met: (1) serial wound cultures were negative for pathogenic bacteria; (2) greater than 90% of the wound bed was covered with healthy granulation tissue; and (3) the patient was hemodynamically stable.

Once these criteria were satisfied, the method of closure was selected based on wound characteristics, including size, depth, location, and the viability of surrounding tissues. Options included delayed primary closure, split-thickness skin grafting, local flap reconstruction, free flap reconstruction, or healing by secondary intention.

Outcome measures

The primary outcome measure was total hospital length of stay, defined as the duration from initial admission to final discharge. Secondary outcome measures included the number of debridement procedures required, time to definitive wound closure (measured from the time of initial debridement to definitive closure), method of definitive closure (delayed primary closure, split-thickness skin grafting, local flap reconstruction, free flap reconstruction, or healing by secondary intention), ambulatory functional decline (defined as worsening of at least one level on the six-point ambulatory scale from premorbid baseline to discharge), and in-hospital mortality.

Additional clinical outcomes assessed included ICU length of stay, duration of mechanical ventilation, total antibiotic treatment duration, and wound-related complications such as delayed healing, infection recurrence, or the need for additional surgical interventions. All outcomes were tracked from the time of initial presentation until hospital discharge or death, whichever occurred first.

Statistical analysis

Categorical variables are presented as frequencies and percentages, and continuous variables are expressed as means with standard deviations for normally distributed data or as medians with interquartile ranges for non-normally distributed data. Normality of distribution was assessed using the Shapiro-Wilk test. Between-group comparisons were performed using the chi-square test or Fisher’s exact test for categorical variables, the unpaired t-test for normally distributed continuous variables, and the Mann-Whitney U test for non-normally distributed continuous variables. A two-sided p-value of less than 0.05 was considered statistically significant. All statistical analyses were performed using EZR (Saitama Medical Center, Jichi Medical University, Saitama, Japan), which is a graphical user interface for R (R Foundation for Statistical Computing, Vienna, Austria) [19].

## Results

Patient characteristics

In total, 74 patients (mean age = 63.61 ± 17.13 years; 70.7% male) were included in the analysis. There were no statistically significant differences (defined as p < 0.05) observed between the NPWT (n = 28) and non-NPWT (n = 46) cohorts for most baseline characteristics, including sex, body mass index, overall comorbidity burden as assessed by the CCI, LRINEC score category, bacteremia rate, and premorbid ambulatory status. However, there was a trend toward a lower age in the NPWT group (mean = 58.82 ± 15.50 vs. 65.93 ± 17.37 years, p = 0.080) and a lower prevalence of cardiovascular disease in the NPWT group (6.7% vs. 24.0%, p = 0.068). Although baseline characteristics were not statistically different between groups, small imbalances in age and cardiovascular comorbidity may still have influenced hospital stay, particularly in this limited sample. Detailed baseline characteristics are presented in Table 1.

**Table 1:** Clinical data. NPWT: negative pressure wound therapy; SD: standard deviation; CKD: chronic kidney disease; COPD: chronic obstructive pulmonary disease; HIV: human immunodeficiency virus; AIDS: acquired immunodeficiency syndrome; LRINEC: Laboratory Risk Indicator for Necrotizing Fasciitis. Statistical significance is set at p < 0.05.

	Non-NPWT	NPWT	Test statistic	p-value
Sample size (n)	46	28	-	-
Age (years, mean ± SD)	65.93 ± 17.37	58.82 ± 15.50	t = 1.78	0.08
Male sex (n, %)	34 (73.9)	19 (67.9)	χ2=0.087	0.604
Body mass index (kg/m^2^, mean ± SD)	25.10 ± 6.13	23.48 ± 5.18	t = 1.15	0.255
Smoke (n, %)	16 (51.6)	10 (47.6)	Fisher's exact	1
Drink (n, %)	28 (60.9)	18 (64.3)	Fisher's exact	0.809
Comorbidities (n, %)	-	-	-	-
Diabetes mellitus	21 (45.7)	11 (39.3)	Fisher's exact	0.635
Cardiovascular	12 (24.0)	2 (6.7)	Fisher's exact	0.068
Malignancy	8 (17.4)	1 (3.6)	Fisher's exact	0.14
Liver disease	2 (4.3)	1 (3.6)	Fisher's exact	1
CKD	6 (13.0)	4 (14.3)	Fisher's exact	1
Collagen disease	1 (2.2)	1 (3.6)	Fisher's exact	1
COPD	1 (2.2)	1 (3.6)	Fisher's exact	1
HIV/AIDS	0 (0)	0 (0)	Fisher's exact	1
Charlson Comorbidity Index (n, %)	-	-	χ2=2.89	0.253
0	14 (30.4)	13 (46.4)	-	-
1	12 (26.1)	8 (28.6)	-	-
≥2	20 (43.5)	7 (25.0)	-	-
LRINEC score (%)	-	-	Fisher's exact	0.495
Low risk	17 (37.0)	9 (32.1)	-	-
Intermediate risk	7 (15.2)	2 (7.1)	-	-
High risk	22 (47.8)	17 (60.7)	-	-
Walking ability before the disease (n, %)	-	-	Fisher's exact	0.739
Walking without assistance	39 (86.7)	23 (82.1)	-	-
Walking with assistance	6 (12.2)	5 (17.9)	-	-
Type of infection(n, %)	-	-	Fisher's exact	1
Monomicrobial	12 (27.9)	7 (25.9)	-	-
Polymicrobial	31 (72.1)	20 (74.1)	-	-
No growth	3 (6.5)	1 (3.6)	-	-
Bacteremia (n, %)	14 (30.4)	14 (50.0)	Fisher's exact	0.138
Affected part (n, %)	-	-	χ2=2.51	0.153
Lower limb	23 (50.0)	19 (67.9)	-	-
Trunk	23 (50.0)	9 (32.1)	-	-

Operative and early management variables

The median time from admission to the first surgical debridement was 7.0 hours (IQR = 4-43 hours) and did not differ significantly between the NPWT and non-NPWT groups (Table 2). Empiric broad-spectrum antibiotics were initiated within a median of three hours in both cohorts, with subsequent culture-guided adjustments made in 82.4% of all cases (Table 2). The NPWT group required a significantly higher number of debridement procedures compared to the non-NPWT group (mean = 2.64 ± 1.66 vs. 1.70 ± 1.30, respectively; p = 0.008). Vasopressor use during the perioperative period and ICU admission rates were comparable between the two groups (Table 2).

**Table 2:** Preoperative treatment data. NPWT: negative pressure wound therapy; IQR: interquartile range. Statistical significance is set at p < 0.05.

	Non-NPWT	NPWT	Test statistic	p-value
Preoperative waiting period (days, median (IQR))	0.00 (0.00-2.00)	0.00 (0.00-2.25)	U=612	0.898
Preoperative waiting period (hours, median (IQR))	7.00 (3.75-48.00)	7.25 (4.88-21.75)	U=509	0.939
Time to first antibiotic administration (hours, median (IQR))	3.00 (2.00-4.38)	3.00 (2.00-4.62)	U=648	0.964
Antibiotic adjustment based on culture results (n, %)	36 (78.3)	25 (89.3)	χ2=0.799	0.347
Use of vasopressors (n, %)	14 (30.4)	11 (40.7)	Fisher's exact	0.447

Wound closure and resource use

As shown in Table 3, the NPWT group underwent more debridement procedures and more frequently required skin grafting than the conventional dressing group. Consequently, the mean hospital length of stay was significantly longer in the NPWT group (80.36 ± 36.84 vs. 43.17 ± 33.63 days; p < 0.001). These findings are summarized in Table 3.

**Table 3:** Treatment outcomes. NPWT: negative pressure wound therapy; SD: standard deviation; ICU: intensive care unit. * Statistical significance is set at p < 0.05.

	Non-NPWT	NPWT	Test statistic	p-value
Total debridement count (n, mean ± SD)	1.70 ± 1.30	2.64 ± 1.66	t=-2.7363	0.008
Time to wound closure (days, mean ± SD)	38.73 ± 45.39	59.07 ± 43.33	U=238	0.083
Last treatment (%)	-	-	Fisher's exact	<0.001*
Skin graft	6 (13.0)	16 (57.1)	-	-
Local flap	1 (2.2)	1 (3.6)	-	-
Free flap	0 (0.0)	2 (7.1)	-	-
Primary wound closure	23 (50.0)	5 (17.9)	-	-
Conservative treatment	16 (34.8)	4 (14.3)	-	-
ICU management period (days, mean ± SD)	2.76 ± 5.26	4.69 ± 5.60	U=443	0.148
Hospitalization period (days, mean ± SD)	43.17 ± 33.63	80.36 ± 36.84	U=241	<0.001*

Functional outcomes

At the time of discharge, the proportion of patients experiencing any decline in ambulatory level compared to their premorbid status was similar between the NPWT group (nine of 28 patients, 33.3%) and the non-NPWT group (13 of 46 patients, 31.0%; p = 1.000). Patients with missing premorbid ambulatory status (NPWT, n = 1; non-NPWT, n = 4) were excluded from the corresponding functional outcome analysis. This finding indicates that the use of NPWT, despite potentially longer treatment periods, did not adversely affect postoperative ambulatory recovery as assessed at discharge (Table 4).

**Table 4:** Patient data at last follow-up. NPWT: negative pressure wound therapy; SD: standard deviation. Statistical significance is set at p < 0.05. Walking ability was assessed using a six-point ordinal scale: 0 = independent ambulation; 1 = cane-assisted or supported walking; 2 = walker or rollator use; 3 = wheelchair; 4 = bedridden; 5 = unknown. Patients with unknown status (score 5) were excluded from the analysis. Walking ability decline was defined as a decrease of at least one level in the walking ability score compared to baseline.

	Non-NPWT	NPWT	Test statistic	p-value
Postoperative complication (n, %)	-	-	Fisher's exact	0.422
Neurological disorders	0 (0.0)	1 (3.8)	-	
Contraction	0 (0.0)	1 (3.8)	-	
Others	2 (4.7)	1 (3.8)	-	
Place of discharge (n, %)	-	-	χ2=4.135	0.463
Home	30 (71.4)	18 (66.7)	-	
Nursing home	2 (4.8)	2 (67.4)	-	
Rehabilitation hospital	4 (9.5)	6 (22.2)	-	
Long-term care hospital	4 (9.5)	1 (3.7)	-	
Unknown	2 (4.8)	0 (0.0)	-	
Final walking ability (n, %)	-	-	χ2=9.661	0.089
0 = Independent ambulation	27 (64.3)	14 (51.9)	-	
1 = Cane-assisted or supported walking	0 (0.0)	4 (14.8)	-	
2 = Walker or rollator use	2 (4.8)	3 (11.1)	-	
3 = Wheelchair	4 (9.5)	3 (11.1)	-	
4 = Bedridden	6 (14.3)	3 (11.1)	-	
5 = Unknown	3 (7.1)	0 (0.0)	-	
Walking ability decline (n, %)	13 (31.0)	9 (33.3)	Fisher's exact	1

To partially address confounding by indication, a propensity score-matched sensitivity analysis was performed. A 1:1 nearest-neighbor matching algorithm was applied using a logistic regression model, incorporating age, sex, BMI, CCI, LRINEC score, cardiovascular disease, diabetes mellitus, hypertension, and premorbid ambulatory status (caliper = 0.1 SD of the logit propensity score). This yielded 18 matched pairs (n = 36 total), achieving acceptable balance on most covariates (standardized mean difference < 0.20 for age, BMI, CCI, LRINEC score, and cardiovascular disease). In the matched cohort, hospital length of stay remained significantly longer in the NPWT group (mean = 87.2 ± 43.6 vs. 34.7 ± 28.4 days; p < 0.001). Split-thickness skin grafting was more frequently required in the NPWT group (50.0% vs. 5.6%; p = 0.007), consistent with the primary analysis. The number of debridement procedures, ambulatory function at discharge, and 60-day mortality did not differ significantly between the matched groups. These results are summarized in Supplementary Table S2.

Survival analysis

**Figure 2:**
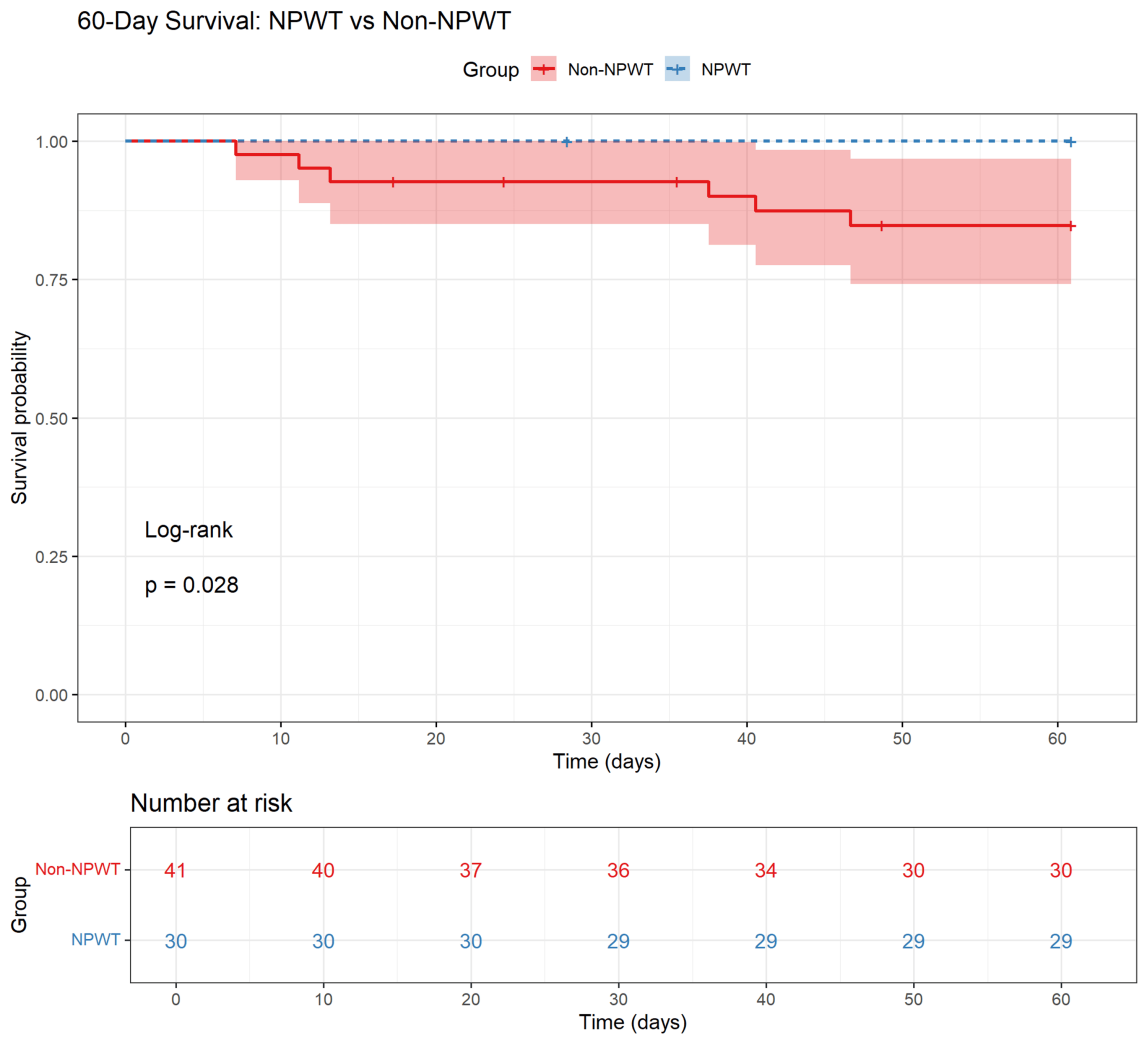
Sixty-day survival probability: NPWT vs. non-NPWT. NPWT: negative pressure wound therapy.

Six deaths occurred within 60 days of admission, all in the non-NPWT group (non-NPWT: 6/41, 14.6%; NPWT: 0/30, 0.0%; Fisher's exact test, p = 0.036). Kaplan-Meier analysis demonstrated a significantly higher 60-day survival probability in the NPWT group (log-rank test, p = 0.028; Figure 2).

The estimated 60-day survival rate was 84.7% (95% CI: 74.2-96.8%) in the non-NPWT group, whereas no deaths were observed in the NPWT group during the 60-day observation period. All six patients who died within 60 days were elderly males (mean age = 82.7 ± 7.4 years) with multiple comorbidities (mean CCI = 3.3 ± 2.1). However, because the NPWT group was younger (mean = 59.7 vs. 65.0 years) and had fewer cardiovascular comorbidities, this difference should be interpreted with caution. Owing to the low event count (n = 6) and complete absence of deaths in the NPWT group, formal multivariable survival analysis was not performed (Supplementary Table S1).

## Discussion

This multicenter retrospective study revealed unexpected findings regarding NPWT use in SSTIs. Despite the theoretical advantages of NPWT in promoting wound healing, patients treated with NPWT experienced significantly longer hospital stays (80.36 vs. 43.17 days, p < 0.001) and required more complex closure methods, with split-thickness skin grafting performed in 57.1% of NPWT cases compared to only 13.0% in the conventional treatment group. These differences in treatment intensity did not translate into improved functional outcomes, as rates of ambulatory recovery were similar between the groups.

The significantly longer hospital stay and higher rate of skin grafting in the NPWT group may reflect fundamental biological and mechanical limitations of NPWT in the context of SSTIs. NPWT applies continuous negative pressure, which mechanically stretches tissues and increases local blood flow through vasodilation and angiogenesis [20]. This mechanical stress upregulates various growth factors, including VEGF, PDGF, and FGF-2, promoting the formation of granulation tissue and epithelialization [21]. However, in SSTIs in which extensive tissue necrosis has occurred, the wound edges often undergo significant retraction and scarring during the inflammatory phase [22].

Critical mechanistic differences emerge during the acute inflammatory phase of severe infections. The massive infiltration of neutrophils and subsequent release of proteases, including elastase and collagenase, create a proteolytic environment that degrades the very growth factors that NPWT aims to concentrate. Matrix metalloproteinases (MMPs), particularly MMP-2 and MMP-9, are markedly elevated in acute severe infections and actively break down extracellular matrix components essential for angiogenesis [22]. Furthermore, the high levels of reactive oxygen species generated during the inflammatory response can denature growth factor proteins, rendering them biologically inactive despite their increased local concentration [23].

The bacterial endotoxins and exotoxins present in SSTIs also interfere with cellular mechanotransduction pathways. These toxins disrupt integrin-mediated cell adhesion and alter cytoskeletal organization, preventing cells from properly responding to the mechanical stimuli provided by negative pressure [24]. Additionally, the profound tissue acidosis (pH often <7.0) common in severe infections impairs cellular metabolism and protein synthesis, limiting the cells’ ability to respond to growth factor signaling despite their presence [25]. The continuous negative pressure may exacerbate this tissue retraction by applying sustained mechanical stress to already compromised wound margins in this hostile biochemical environment [26].

The pathophysiology of wound healing in SSTIs fundamentally differs from that of other conditions. Previous trials predominantly focused on diabetic foot ulcers and chronic wounds characterized by impaired healing [27,28]. In contrast, necrotizing fasciitis and gas gangrene involve acute bacterial invasion with rapid tissue destruction. Bacterial toxins from Group A *Streptococcus* and *Clostridium* species cause direct endothelial damage and microvascular thrombosis, creating conditions that may render the mechanical stimulation of NPWT ineffective. Moreover, these infections create large, irregular defects with compromised vascular supply, where negative pressure distribution may be uneven and potentially harmful.

Despite significant differences in hospital stay and closure complexity, ambulatory function at discharge was similar between the two groups (33.3% vs. 31.0% decline, p = 1.000). Functional recovery in SSTIs is primarily determined by initial tissue destruction rather than subsequent wound management. This equivalence in functional outcomes, combined with prolonged hospitalization and increased costs, strengthens the argument against routine NPWT use in this population.

Several important limitations should be acknowledged. First, necrotizing soft-tissue infections are clinically heterogeneous, and the two treatment groups may not have been fully comparable. NSTIs vary in anatomical site, extent of necrosis, wound depth, microbiology, systemic severity, and timing of source control. In addition, detailed quantitative indicators of wound severity, such as wound surface area, depth, and standardized assessments of tissue viability, were not consistently recorded across institutions. This limited our ability to accurately assess wound severity and fully adjust for confounding factors. Accordingly, these findings should be interpreted with caution, and the results should be considered as associations rather than causal relationships. To partially address this limitation, we conducted a propensity score-matched sensitivity analysis (18 matched pairs), which confirmed the primary findings regarding hospital stay and wound closure methods. However, important residual confounding remains plausible, as key wound-severity measures were unavailable for matching. Future prospective studies incorporating standardized and objective wound assessments are warranted to better clarify the comparative effectiveness of NPWT in this population.

Second, the study did not capture variations in NPWT application techniques, including foam versus gauze interface selection, pressure setting modifications beyond the reported ranges (-80 to -125 mmHg), dressing change frequency, or duration of therapy before closure attempts. These technical factors may significantly influence NPWT effectiveness. Fourth, our outcome measurement approach, while comprehensive in assessing the entire treatment trajectory from initial debridement to discharge, differs from most published NPWT studies that measure from therapy initiation. Though this methodological difference provides a valuable clinical perspective, it limits direct comparison with the existing literature. Finally, the single-country, predominantly academic medical center setting may limit generalizability to other healthcare systems with different resource availability, surgical expertise, or patient populations.

This study challenges routine NPWT application in SSTIs. NPWT use was associated with prolonged hospitalization and increased procedural complexity without functional benefits. It is also important to consider the context of the healthcare system in which this study was conducted. In Japan, early discharge to home or subacute care facilities with ongoing NPWT or daily dressing changes is not routinely practiced due to limitations in outpatient wound care infrastructure and reimbursement policies. Therefore, patients often remain hospitalized until complete wound closure is achieved. This practice likely contributed to the prolonged length of hospital stay observed in both treatment groups and may limit direct comparison with international data, where outpatient NPWT is more commonly employed.

Given the substantial resource implications and lack of demonstrated superiority in this retrospective cohort, routine use of NPWT in all SSTI cases should be approached with caution. Future prospective studies are needed to identify patient subgroups who may benefit most from NPWT based on wound characteristics and comorbid conditions.

## Conclusions

In this multicenter retrospective study, NPWT use for necrotizing fasciitis or gas gangrene of the lower extremity and trunk was associated with a significantly longer hospital stay (mean = 80.4 vs. 43.2 days; p < 0.001) and more frequent split-thickness skin grafting. These findings were consistent in a propensity score-matched sensitivity analysis. All six deaths within 60 days occurred in the non-NPWT group; however, this observation should be interpreted cautiously, given baseline differences in age and cardiovascular comorbidity and the low event count. Functional outcomes at discharge were comparable between groups.

Given that NPWT selection was not standardized and likely reflected surgeon discretion, selection bias may have influenced the observed outcomes. NPWT may serve as a useful adjunct for local wound management without compromising early functional recovery; however, its routine use should be approached with caution. Prospective studies incorporating standardized wound severity assessment and clear criteria for NPWT initiation are warranted to identify patients most likely to benefit.

## Appendices

Supplementary Table S1A

**Table 5:** Univariate Cox proportional hazards analysis for 60-day mortality. N = 71 patients; 6 events (NPWT group: 0/30 deaths; conventional dressing group: 6/41 deaths). HR: hazard ratio; CI: confidence interval; NPWT: negative pressure wound therapy; CCI: Charlson Comorbidity Index; CCI_ge2: Charlson Comorbidity Index ≥ 2; preADL_dep: pre-admission dependence in activities of daily living; DM: diabetes mellitus; Cardio: cardiovascular disease; LRINEC: Laboratory Risk Indicator for Necrotizing Fasciitis; LRINEC_high: high-risk category of LRINEC; HT: hypertension; BMI: body mass index. Firth penalized: Firth's penalized partial likelihood (coxphf R package v1.13.4); 95% CI by profile likelihood. Standard Cox (survival::coxph, Efron ties) was used for all other variables. Sorted by P-value. Continuous: age (per year), BMI (per kg/m²), CCI, and LRINEC.

Variable	HR (95% CI)	P-value	Note
Age	1.126 (1.034-1.226)	0.006	
NPWT	0.096 (0.001-0.812)	0.028	Firth penalized
CCI ≥2	9.357 (1.093-80.103)	0.041	
CCI	1.173 (0.949-1.450)	0.140	
Male	5.232 (0.618-682.441)	0.153	Firth penalized
preADL_dep	2.396 (0.439-13.086)	0.313	
DM	2.360 (0.432-12.888)	0.321	
Cardio	1.851 (0.339-10.110)	0.477	
LRINEC	1.105 (0.836-1.461)	0.482	
HT	1.492 (0.301-7.393)	0.624	
LRINEC ≥8	0.735 (0.148-3.640)	0.706	
BMI	0.976 (0.828-1.152)	0.777	

Supplementary Table S1B

**Table 6:** Multivariate Firth-penalized Cox regression for 60-day mortality. Covariates: Age + NPWT (pre-specified exploratory model; N = 71, events = 6). HR: hazard ratio; CI: confidence interval; NPWT: negative pressure wound therapy. Firth-penalized partial likelihood applied to both covariates (coxphf R package v1.13.4); 95% CI by profile likelihood. With six events (events per variable (EPV) constraint), a two-covariate model (Age + NPWT) was pre-specified as an exploratory analysis. The NPWT group had 0 deaths within 60 days (complete separation); Firth penalization stabilizes the NPWT coefficient.

Variable	HR (95% CI)	P-value	Note
Age	1.091 (1.022-1.194)	0.006	Firth penalized
NPWT	0.169 (0.001-1.592)	0.138	Firth penalized

Supplementary Table S2

**Table 7:** Clinical outcomes after propensity score matching comparing NPWT and conventional dressing groups. LOS: length of stay; NPWT: negative pressure wound therapy; STSG: split-thickness skin grafting; ADL: activities of daily living.

Outcome	NPWT group	Non-NPWT group	P-value	Test
LOS (days), mean ± SD (NPWT, n = 18; Non-NPWT, n = 18)	87.2 ± 43.6	34.7 ± 28.4	0.0001	Wilcoxon
Debridements (times), mean ± SD	2.6 ± 1.7	1.8 ± 1.3	0.2006	Wilcoxon
Skin grafting (STSG), n (%)	9 (50%)	1 (5.6%)	0.0072	Fisher
Primary closure, n (%)	4 (22.2%)	10 (55.6%)	0.0858	Fisher
ADL decline at discharge, n (%)	6 (35.3%)	2 (14.3%)	0.2399	Fisher
60-day mortality, n (%)	0 (0%)	4 (22.2%)	0.1039	Fisher

## References

[1] Green RJ, Dafoe DC, Raffin TA (1996). Necrotizing fasciitis. Chest.

[2] Bellapianta JM, Ljungquist K, Tobin E, Uhl R (2009). Necrotizing fasciitis. J Am Acad Orthop Surg.

[3] Leiblein M, Wagner N, Adam EH, Frank J, Marzi I, Nau C (2020). Clostridial gas gangrene - a rare but deadly infection: case series and comparison to other necrotizing soft tissue infections. Orthop Surg.

[4] Puvanendran R, Huey JC, Pasupathy S (2009). Necrotizing fasciitis. Can Fam Physician.

[5] Headley AJ (2003). Necrotizing soft tissue infections: a primary care review. Am Fam Physician.

[6] Guliyeva G, Huayllani MT, Sharma NT, Janis JE (2024). Practical review of necrotizing fasciitis: principles and evidence-based management. Plast Reconstr Surg Glob Open.

[7] Machado NO (2011). Necrotizing fasciitis: the importance of early diagnosis, prompt surgical debridement and adjuvant therapy. N Am J Med Sci.

[8] Hsiao GH, Chang CH, Hsiao CW, Fanchiang JH, Jee SH (1998). Necrotizing soft tissue infections. Surgical or conservative treatment?. Dermatol Surg.

[9] Chen SJ, Chen YX, Xiao JR, Wei XZ, Chen SM, Jiang WZ (2019). Negative pressure wound therapy in necrotizing fasciitis of the head and neck. J Oral Maxillofac Surg.

[10] de Paula FM, Pinheiro EA, Oliveira VM, Ferreira CM, Monreal MT, Rolan MD, Matos VT (2019). A case report of successful treatment of necrotizing fasciitis using negative pressure wound therapy. Medicine (Baltimore).

[11] Livingstone JP, Hasegawa IG, Murray P (2018). Utilizing negative pressure wound therapy with instillation and dwell time for extensive necrotizing fasciitis of the lower extremity: a case report. Cureus.

[12] Corona PS, Erimeiku F, Reverté-Vinaixa MM, Soldado F, Amat C, Carrera L (2016). Necrotising fasciitis of the extremities: implementation of new management technologies. Injury.

[13] Lipatov KV, Asatryan A, Melkonyan G (2024). Effectiveness of negative pressure wound therapy in complex surgical treatment of necrotizing fasciitis of the upper limb. World J Orthop.

[14] Czymek R, Schmidt A, Eckmann C (2009). Fournier's gangrene: vacuum-assisted closure versus conventional dressings. Am J Surg.

[15] Stevens DL, Bisno AL, Chambers HF (2014). Practice guidelines for the diagnosis and management of skin and soft tissue infections: 2014 update by the Infectious Diseases Society of America. Clin Infect Dis.

[16] Costa ML, Achten J, Bruce J, Tutton E, Petrou S, Lamb SE, Parsons NR (2018). Effect of negative pressure wound therapy vs standard wound management on 12-month disability among adults with severe open fracture of the lower limb: the WOLLF randomized clinical trial. JAMA.

[17] Quan H, Sundararajan V, Halfon P (2005). Coding algorithms for defining comorbidities in ICD-9-CM and ICD-10 administrative data. Med Care.

[18] Wong CH, Khin LW, Heng KS, Tan KC, Low CO (2004). The LRINEC (Laboratory Risk Indicator for Necrotizing Fasciitis) score: a tool for distinguishing necrotizing fasciitis from other soft tissue infections. Crit Care Med.

[19] Kanda Y (2013). Investigation of the freely available easy-to-use software 'EZR' for medical statistics. Bone Marrow Transplant.

[20] Daigle P, Despatis MA, Grenier G (2013). How mechanical deformations contribute to the effectiveness of negative-pressure wound therapy. Wound Repair Regen.

[21] Ravindhran B, Schafer N, Howitt A, Carradice D, Smith G, Chetter I (2023). Molecular mechanisms of action of negative pressure wound therapy: a systematic review. Expert Rev Mol Med.

[22] Landén NX, Li D, Ståhle M (2016). Transition from inflammation to proliferation: a critical step during wound healing. Cell Mol Life Sci.

[23] Checa J, Aran JM (2020). Reactive oxygen species: drivers of physiological and pathological processes. J Inflamm Res.

[24] Cheng B, Liu Y, Zhao Y (2019). The role of anthrax toxin protein receptor 1 as a new mechanosensor molecule and its mechanotransduction in BMSCs under hydrostatic pressure. Sci Rep.

[25] Torres IM, Demirdjian S, Vargas J, Goodale BC, Berwin B (2017). Acidosis increases the susceptibility of respiratory epithelial cells to Pseudomonas aeruginosa-induced cytotoxicity. Am J Physiol Lung Cell Mol Physiol.

[26] Torbrand C, Anesäter E, Borgquist O, Malmsjö M (2018). Mechanical effects of negative pressure wound therapy on abdominal wounds - effects of different pressures and wound fillers. Int Wound J.

[27] Liu Z, Dumville JC, Hinchliffe RJ (2018). Negative pressure wound therapy for treating foot wounds in people with diabetes mellitus. Cochrane Database Syst Rev.

[28] Burhan A, Khusein NB, Sebayang SM (2022). Effectiveness of negative pressure wound therapy on chronic wound healing: a systematic review and meta-analysis. Belitung Nurs J.

